# Iterative framework for the joint segmentation and CT synthesis of MR images: application to MRI-only radiotherapy treatment planning

**DOI:** 10.1088/1361-6560/aa66bf

**Published:** 2017-05-05

**Authors:** Ninon Burgos, Filipa Guerreiro, Jamie McClelland, Benoît Presles, Marc Modat, Simeon Nill, David Dearnaley, Nandita deSouza, Uwe Oelfke, Antje-Christin Knopf, Sébastien Ourselin, M Jorge Cardoso

**Affiliations:** 1 Translational Imaging Group, CMIC, University College London, London, United Kingdom; 2 Department of Radiotherapy, University Medical Center Utrecht, Utrecht, Netherlands; 3 Centre for Medical Image Computing, University College London, London, United Kingdom; 4 Dementia Research Centre, Institute of Neurology, UCL, London, United Kingdom; 5 Joint Department of Physics, Institute of Cancer Research and Royal Marsden NHS Foundation Trust (ICR/RMH), London, United Kingdom; 6 Academic Urology Unit, ICR/RMH, Sutton, United Kingdom; 7 CRUK Centre for Cancer Imaging, ICR/RMH, Sutton, United Kingdom; 8 University of Groningen, University Medical Center Groningen, Department of Radiation Oncology, Groningen, Netherlands; n.burgos@ucl.ac.uk

**Keywords:** segmentation, image synthesis, atlas-based methods, pseudo CT, MRI-only RTP

## Abstract

To tackle the problem of magnetic resonance imaging (MRI)-only radiotherapy treatment planning (RTP), we propose a multi-atlas information propagation scheme that jointly segments organs and generates pseudo x-ray computed tomography (CT) data from structural MR images (T1-weighted and T2-weighted). As the performance of the method strongly depends on the quality of the atlas database composed of multiple sets of aligned MR, CT and segmented images, we also propose a robust way of registering atlas MR and CT images, which combines structure-guided registration, and CT and MR image synthesis.

We first evaluated the proposed framework in terms of segmentation and CT synthesis accuracy on 15 subjects with prostate cancer. The segmentations obtained with the proposed method were compared using the Dice score coefficient (DSC) to the manual segmentations. Mean DSCs of 0.73, 0.90, 0.77 and 0.90 were obtained for the prostate, bladder, rectum and femur heads, respectively. The mean absolute error (MAE) and the mean error (ME) were computed between the reference CTs (non-rigidly aligned to the MRs) and the pseudo CTs generated with the proposed method. The MAE was on average $45.7\pm 4.6$ HU and the ME $-1.6\pm 7.7$ HU. We then performed a dosimetric evaluation by re-calculating plans on the pseudo CTs and comparing them to the plans optimised on the reference CTs. We compared the cumulative dose volume histograms (DVH) obtained for the pseudo CTs to the DVH obtained for the reference CTs in the planning target volume (PTV) located in the prostate, and in the organs at risk at different DVH points. We obtained average differences of $-0.14 \% $ in the PTV for ${{D}_{98 \% }}$, and between $-0.14 \% $ and 0.05% in the PTV, bladder, rectum and femur heads for *D*_mean_ and ${{D}_{2 \% }}$.

Overall, we demonstrate that the proposed framework is able to automatically generate accurate pseudo CT images and segmentations in the pelvic region, potentially bypassing the need for CT scan for accurate RTP.

## 1. Introduction

The aim of radiotherapy treatment planning (RTP) is to optimise the therapeutic ratio by delivering an optimal dose of radiation over the target area while sparing the normal tissues. RTP first requires contouring the target and organs at risk (OARs). Once these volumes have been defined, the attenuation properties of the different tissues are used as parameters in an optimisation process calculating the optimal dose distribution to treat the tumour. Most radiotherapy treatments are planned using an x-ray computed tomography (CT) scan of the patient. The acquisition of a CT is fast and the tissue attenuation coefficients can easily be derived from the CT intensity values in Hounsfield unit (HU). However, CT images have a low soft tissue contrast, which can lead to organ delineation errors, particularly when located in the brain, head & neck, or pelvic regions (Rasch *et al* 2005). Magnetic resonance (MR) imaging is often preferred over CT as a structural imaging modality, mainly for its excellent soft tissue contrast. Although MR is increasingly used in clinical practice, its role in RTP is currently limited by the fact that it does not readily provide electron density information, hampering the calculation of dose distributions. This is a critical limitation for the clinical deployment of the new devices combining an MR scanner and a linear accelerator (MR-linac).

In this work, we propose to tackle the problem of RTP from MR images by developing a multi-atlas propagation framework to estimate the tissue attenuation properties and jointly delineate the organs of interest. Although the principal target of this work is RTP, mainly to facilitate the clinical deployment of MR-linac devices, the methods developed are also relevant to the attenuation correction of positron emission tomography (PET) images acquired on hybrid PET/MR scanners (Izquierdo-Garcia and Catana 2016).

Multi-atlas propagation was first introduced for segmentation purposes (Heckemann *et al* 2006, Klein *et al* 2008, Cabezas *et al* 2011). The technique relies on a database of pairs of intensity and segmented images, often called ‘atlases’. To segment the target image, a first step consists of registering the atlas intensity images to the target intensity image, and to apply the same transformations to the associated segmented images. A second step consists of fusing the propagated segmented images to generate the target segmentation. The technique was later extended to the synthesis of images by propagating intensity images instead of segmented images, for example CT images (Burgos *et al* 2014). Many multi-atlas CT synthesis methods have been developed for RTP (Gudur *et al* 2014, Uh *et al* 2014, Burgos *et al* 2015, Dowling *et al* 2015, Sjölund *et al* 2015, Arabi *et al* 2016) but only a few have been applied outside of the brain (Burgos *et al* 2015, Dowling *et al* 2015, Arabi *et al* 2016).

Even though atlas-based segmentation and CT synthesis methods have been successfully applied to RTP independently, a key to expand MR-based planning is to guarantee that the segmentations and pseudo CT generated from the MR images match each other, i.e. a voxel labelled as bone should have a bone density value in the pseudo CT image. This is not guaranteed if the segmentation and CT synthesis tasks are handled separately. Dowling *et al* (2012) proposed to combine CT synthesis and segmentation using a single atlas composed of an MR, a CT and a segmented image obtained via groupwise registration. The target CT and segmented images were obtained by registering the atlas MR image to the target MR image and applying the same transformation to the atlas CT and segmented images. The accuracy of single-atlas methods is limited as a single atlas can hardly be representative of all the potential targets and because they strongly depend on the quality of the registration used to map the atlas to the target subject. Dowling and colleagues then extended the method to multiple atlases (Dowling *et al* 2015), each atlas being composed of an MR, a CT and a segmented image. Similarly to their previous method, the atlas MR images were first registered to the target MR image and the same transformations were applied to both the atlas CT and segmented images. A locally weighted voting method was then used to generate the target CT and segmented images.

In this paper, we develop an iterative multi-atlas propagation framework that combines in a single pipeline segmentation and CT synthesis, with the aim to improve both the segmentation and synthesis accuracies when compared to state-of the art methods, and guarantee consistent results. We also propose a new strategy to register atlas MR and CT images that combines structure-guided registration and image synthesis, with the aim to build a higher quality atlas database and thus further improve the segmentation and synthesis accuracies. This paper is an extension of preliminary work (Burgos *et al* 2016a, 2016b).

## 2. Materials and methods

In this section, we present the data used to develop and validate the proposed methods (section 2.1), describe the proposed iterative multi-atlas propagation framework (section 2.2), detail how the multi-atlas database was built (section 2.3) and explain our validation strategy (section 2.4).

### 2.1. Data acquisition and preprocessing

The proposed framework was evaluated on a retrospective study comprising 15 prostate cancer patients treated with fixed-field intensity-modulated therapy (prescribed dose range, 67–82 Gy). All patients included in this study had given consent for their data to be used for research purposes. Each subject had a T2-weighted MR image (3 T, 2D spin echo; TE/TR: 80/2500 ms; $1.46\times 1.46\times 5~\text{m}{{\text{m}}^{3}}$), a T1-weighted MR image (3 T, 2D spin echo; TE/TR: 10/400 ms; $1.64\times 1.64\times 5~\text{m}{{\text{m}}^{3}}$), and a CT image (140 kVp, voxel size $0.98\times 0.98\times 1.5~\text{m}{{\text{m}}^{3}}$), all acquired the same day. Delineations of the organs were performed manually by a qualified clinician for each modality independently. Note that a different couch was used for the MR (curved couch) and CT (flat couch) imaging sessions. Image preprocessing consisted of resampling the MR images to isotropic resolution using a cubic spline interpolation, and performing intensity non-uniformity correction (Tustison *et al* 2010).

### 2.2. Joint iterative segmentation and image synthesis

The proposed iterative framework relies on a multi-atlas database consisting for each atlas of a T2-weighted and a T1-weighted MR image, a CT image, and a manually segmented image, all co-registered (details presented in section 2.3). Both the T2-weighted and T1-weighted MR images are used as inputs for the method since they provide complementary information to describe the subject’s anatomy (Burgos *et al* 2015a).

At the initial iteration, a set of probabilistic segmentations and pseudo CT (pCT) image is jointly generated from the target’s MR images by registering the atlas MR images to the target, and fusing the propagated atlas segmentations and CT images according to the similarity between each atlas MR images and the target MR images.

For the subsequent iterations, similarly to Bai *et al* (2013), the previously generated set of probabilistic segmentations and pCT images is combined with the target MR images. A new refined set of probabilistic segmentations and pCT images is jointly generated from the target’s MR images, and the previous set of probabilistic segmentations and pCT image, first by registering the atlas MR, CT and segmented images to the target. The propagated atlas segmentations and CT images are then fused according to the similarity between each atlas MR, CT and segmented images, and the target MR images and previous set of probabilistic segmentations and pCT images.

**Figure 1.**
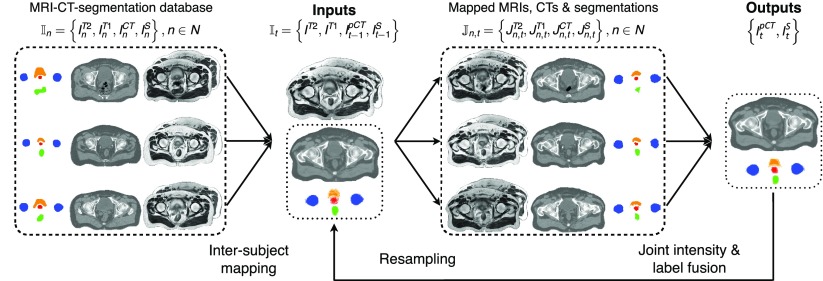
Joint segmentation and CT synthesis at iteration *t*. All the atlases are non-rigidly registered to the target. A local similarity measure between the mapped atlases and the target is used to jointly generate a pseudo CT and a segmented image.

A diagram illustrating the proposed method is shown in figure 1.

#### 2.2.1. Joint iterative label and intensity fusion.

Let the target subject’s dataset at iteration *t* be denoted by ${{\mathbb{I}}_{t}}=\left\{{{I}^{T2}},{{I}^{T1}},I_{t-1}^{\text{pCT}},I_{t-1}^{S}\right\}$ where *I*^*T*2^ and *I*^*T*1^ are the T2- and T1-weighted MR images, and $I_{t-1}^{\text{pCT}}$ and $I_{t-1}^{S}$ are the pCT and segmented images obtained at iteration *t*  −  1. Let the dataset of the *n*th atlas in the database be denoted by ${{\mathbb{I}}_{n}}=\left\{I_{n}^{T2},I_{n}^{T1},I_{n}^{\text{CT}},I_{n}^{S}\right\}$ where $I_{n}^{\text{CT}}$ corresponds to the real CT and $I_{n}^{S}$ to the manual segmentation.

The first step of the method consists of registering each atlas to the target subject. This inter-subject coordinate mapping was obtained using a symmetric global registration followed by a cubic B-spline parametrised non-rigid multi-channel registration as implemented in NiftyReg (Modat *et al* 2010). The local normalised cross-correlation (LNCC) was used as a similarity measure for the MR and CT channels, and the Kullback–Leibler divergence (KLD) for the segmentation channels. The non-rigid registrations were performed with a pyramidal approach with five levels. The finer lattice of control points had a spacing of 10 mm along each axis. A linear interpolation was used during the optimisation.

At each iteration *t*, a new transformation ${{\mathcal{T}}_{n,t}}$ that maps atlas *n* to the target was defined, generating a set of images aligned to the target: ${{\mathbb{J}}_{n,t}}=\left\{J_{n,t}^{T2},J_{n,t}^{T1},J_{n,t}^{\text{CT}},J_{n,t}^{S}\right\}$ where $J_{n,t}^{M}(x)=I_{n}^{M}\left({{\mathcal{T}}_{n,t}}(x)\right)$ with $M=\left\{T1,T2,CT,S\right\}$ and *x* a voxel.

The pCT and probabilistic segmentations were then obtained by fusing the *N* atlases mapped to the target subject as follows:

1 \begin{eqnarray*}I_{t}^{\text{pCT}}(x)=\frac{\underset{n=1}{\overset{N}{\sum}}\,{{w}_{n,t}}(x)\centerdot J_{n,t}^{\text{CT}}(x)}{\underset{n=1}{\overset{N}{\sum}}\,{{w}_{n,t}}(x)},\end{eqnarray*}

2 \begin{eqnarray*}I_{t}^{S}(x,l)=\frac{\underset{n=1}{\overset{N}{\sum}}\,{{w}_{n,t}}(x)\centerdot {{V}_{n,t}}(x,l)}{\underset{k=1}{\overset{L}{\sum}}\,\underset{n=1}{\overset{N}{\sum}}\,{{w}_{n,t}}(x)\centerdot {{V}_{n,t}}(x,k)}.\end{eqnarray*}

*l* indexes through the labels and *L* is the number of all possible labels. *V*_*n*,*t*_(*x*,*l*) is the vote for label *l* produced by the *n*th atlas at voxel *x* (Wang *et al* 2013)

3 \begin{eqnarray*}{{V}_{n,t}}(x,l)=\left\{\begin{array}{*{35}{l}} 1 &amp; \text{if}~J_{n,t}^{S}(x,l)=l,l\in \left\{1..L\right\} \\ 0 &amp; \text{otherwise} \end{array}.\right. \end{eqnarray*}

If required, the categorical label result $\mathcal{L}$ at location *x* can thus be obtained by estimating $\mathcal{L}(x)={{\max}_{l}}\left({{I}^{S}}(x,l)\right)$.

The weighting factor *w*_*n*,*t*_(*x*) was obtained by applying an exponential decay function to the rank *r*_*n*,*t*_(*x*) of the local image similarity measure (LSIM, described in the next section) used to assess the similarity between atlas *n* and the target, at each voxel *x* (Burgos *et al* 2014)

4 \begin{eqnarray*}{{w}_{n,t}}(x)={{\text{e}}^{-{{\beta}_{t}}{{r}_{n,t}}(x)}}.\end{eqnarray*}

After each iteration, the registration for all the atlases improves and more atlases can contribute to the fusion. As a smaller *β* means that more atlases contribute to the average, we set *β* to decrease with the number of iterations (by 0.125 starting from ${{\beta}_{1}}=1$).

Note that at the first iteration both the inter-subject mapping and fusion steps were based on the MR images only.

#### 2.2.2. Convolution-based local similarity measures.

To locally select the atlases used in the fusion, a combination of two similarity measures computed between the target and atlases was used. The structural similarity (Wang *et al* 2004) extended to irregular regions-of-interest (ROI) was computed on the MR and CT channels. For the MR channels, the similarity was measured between each mapped atlas MR image and the target MR image, while for the CT channel, the similarity was measured between each mapped atlas CT image and the target pCT image obtained at the previous iteration. The ROI-SSIM between images *I* and *J* at voxel *x* is given by

5 \begin{eqnarray*}\text{ROI-SSIM}\left(I(x),J(x)\right)=\frac{2{{\mu}_{I}}(x){{\mu}_{J}}(x)+{{C}_{1}}}{\mu _{I}^{2}(x)+\mu _{J}^{2}(x)+{{C}_{1}}}\frac{2{{\sigma}_{I,J}}(x)+{{C}_{2}}}{\sigma _{I}^{2}(x)+\sigma _{J}^{2}(x)+{{C}_{2}}}.\end{eqnarray*}

*C*_1_ and *C*_2_ are two constants used to improve the stability of the structural similarity (Wang *et al* 2004). Let $ \Omega $ be a density function equal to 1 where the fields of view (FOV) overlap, and 0 otherwise. The means and standard deviations at voxel *x* were calculated using a Gaussian kernel ${{G}_{{{\sigma}_{G}}}}$ with standard deviation ${{\sigma}_{G}}$ through density normalised convolution (Cachier *et al* 2003)

\begin{eqnarray*}\begin{array}{c} {{\mu}_{I}}(x)=\frac{\left[{{G}_{{{\sigma}_{G}}}}\ast I\right](x)}{\left[{{G}_{{{\sigma}_{G}}}}\ast \Omega \right](x)}, &amp; \sigma _{I}^{2}(x)={{\mu}_{{{I}^{2}}}}(x)-\mu _{I}^{2}(x), &amp; {{\sigma}_{I,J}}(x)={{\mu}_{I\centerdot J}}(x)-{{\mu}_{I}}(x)\centerdot {{\mu}_{J}}(x), \end{array}\end{eqnarray*}

where $\ast $ denotes the convolution operator and ${{G}_{{{\sigma}_{G}}}}\ast \Omega $ represents a density normalisation term that compensates for areas with missing information. We set ${{\sigma}_{G}}=3$ (Burgos *et al* 2014). As the values of ROI-SSIM are only valid within the bounds of the FOV, values outside the FOV were set to $-\infty $.

A local fuzzy (Zadeh 1965) Dice score coefficient (DSC) defined per label *l* and summed over all labels was used to assess the local overlap between the probabilistic segmented images *I*^*S*^ and *J*^*S*^

6 \begin{eqnarray*}\text{LDSC}\left({{I}^{S}}(x),{{J}^{S}}(x)\right)=\underset{l\in \left\{1..L\right\}}{\sum}\,\frac{2\min \left({{\mu}_{{{I}^{S}}}}(x,l),{{\mu}_{{{J}^{S}}}}(x,l)\right)}{{{\mu}_{{{I}^{S}}}}(x,l)+{{\mu}_{{{J}^{S}}}}(x,l)}.\end{eqnarray*}

${{\mu}_{{{I}^{S}}}}(x,l)$ was obtained by convolving the segmentation density *I*^*S*^ for each label with the Gaussian kernel ${{G}_{{{\sigma}_{G}}}}$: ${{\mu}_{{{I}^{S}}}}(x,l)=\left[{{G}_{{{\sigma}_{G}}}}\ast {{I}^{S}}\right](x,l)$.

The final local similarity measure (LSIM) computed at iteration *t* between the target and the *n*th atlas was obtained by summing the ROI-SSIM computed on the MR and CT channels and the LDSC computed on the segmentation channel for each voxel *x*

7 \begin{eqnarray*}\begin{array}{*{35}{l}} \text{LSIM}\left({{\mathbb{I}}_{t-1}}(x),{{\mathbb{J}}_{n,t}}(x)\right)=\text{ROI-SSIM}\left({{I}^{T2}}(x),J_{n,t}^{T2}(x)\right)+\text{ROI-SSIM}\left({{I}^{T1}}(x),J_{n,t}^{T1}(x)\right) \\ \qquad \qquad \qquad \qquad \quad \\ \ +\,\text{ROI-SSIM}\left(I_{t-1}^{\text{pCT}}(x),J_{n,t}^{\text{CT}}(x)\right)+\text{LDSC}\left(I_{t-1}^{S}(x),J_{n,t}^{S}(x)\right). \end{array}\end{eqnarray*}

### 2.3. Atlas database building

The performance of the method presented in the previous section strongly depends on the quality of the multi-atlas database: for each atlas, the T2- and T1-weighted MR images, and the CT image have to be well aligned. Registering MR and CT images is a challenging task, especially in body regions such as the pelvis where large morphological differences can be observed, for example due to different acquisition protocols (different couches), or time differences between the two MR and CT acquisitions resulting in different bladder or rectum filling. To improve multi-modal MR-CT registration, Dowling *et al* (2015) exploited the manual contours delineated on both the MR and CT images to perform structure-guided deformable registration (Rivest-Hénault *et al* 2013). This strategy showed an improved alignment between the MR and CT images but does not entirely overcome the limitations of multi-modal registration. Another strategy to improve multi-modal registration is to reduce the problem to monomodal registration using image synthesis (Iglesias *et al* 2013, Roy *et al* 2014, Chen *et al* 2015, Cao *et al* 2016). In the context of MR-CT registration, the idea consists of synthesising a pseudo CT image from an MR image using co-registered pairs of MR and CT images as atlases. The pseudo CT is then registered to the original CT using a non-rigid registration and the resulting transformation is applied to the MR image.

In this work we propose to combine structure-guided registration and image synthesis to register for each subject the MR and CT images and build the multi-atlas database. Structure-guided registration was used to register each subject’s CT and T1 images to their T2 image, and thus create an initial database. Then, to improve the CT to T2 mapping, for each subject in the database, pseudo CT and pseudo T2 images were generated from the subject’s T2 and CT images, respectively. These pseudo CT and pseudo T2 images, together with the real CT and T2 images, and the segmented images, were used to map CT and T2 spaces.

#### 2.3.1. Initial atlas database.

The initial atlas database was composed of three image-segmentation pairs (ISPs) per subject, one for each data type (T1, T2, CT). The T1 and CT ISPs were registered to the T2 ISP using an affine followed by a multi-channel non-rigid registration (Modat *et al* 2012). The similarity measure used to non-rigidly register two ISPs was defined as the LNCC over the intensity data and KLD over the segmentations, thus aligning both imaging and segmentation data, similarly to Dowling *et al* (2015). All the non-rigid registrations were performed with a pyramidal approach with three levels. The finer lattice of control points had a spacing of 2.5 mm along each axis for the T1 to T2 registrations and 7.5 mm along each axis for the CT to T2 registrations. A linear interpolation was used during the optimisation. After all registrations, the three ISPs per subject were aligned to each other.

#### 2.3.2. Refined atlas database.

Using a leave-one-out strategy, the method in Burgos *et al* (2014) was used to generate a pCT by registering the T2 images from a T2-CT database to the target T2, and propagating and fusing the CT images. The similarity metric used to fuse the propagated CT images was the ROI-SSIM.

Also using a leave-one-out strategy, the method in Burgos *et al* (2014) was again used to generate a pseudo T2 (pT2) by registering the CT images from a CT-T2 database to the target CT.

**Figure 2.**
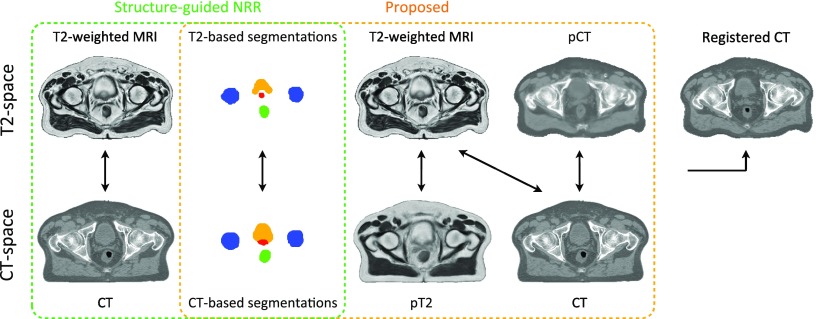
Inputs of the multi-channel registration used to align for each atlas the CT and T2-weighted MR images, and create the initial (green) and refined (orange) atlas database. Note that the pseudo CT (pCT) was generated from the T2-weighted MR image and the pseudo T2 (pT2) from the CT image.

After generating the pCT and pT2, we improved the CT to T2 registration by registering the set {T2, T2_seg_, pCT} with the set {pT2, CT_seg_, CT}, using the LNCC between imaging channels and the KLD between segmentation channels. Note that the similarity term between the T2 and CT channels was preserved to account for multi-modal correlation terms. The non-rigid registrations were performed with a pyramidal approach with three levels. The finer lattice of control points had a spacing of 7.5 mm along each axis. The new coordinate mapping between CT and T2 was used to update the CT alignment to the T2 space. The images used as inputs to align for each atlas the CT and T2-weighted MR images, and create the initial and refined atlas databases are displayed in figure 2.

The refined multi-atlas database consisted for each atlas of a T2-weighted MR image, a T2-based segmented image, a T1-weighted MR image and a CT image, all co-registered. The number of atlases was artificially increased by left-right flipping the images.

### 2.4. Evaluation strategy

#### 2.4.1. Multi-modal registration for atlas database building.

To assess the benefits of the proposed multi-modal T2-CT registration strategy, atlas CT and T2 images were also registered using the structure-guided registration method used to build the initial database (*structure-guided NRR*, see section 2.3.1), using NiftyReg without structure guidance (*NRR*) (Modat *et al* 2012), and using a symmetric affine registration (*affine*) (Modat *et al* 2014). The quality of the registrations was first assessed visually. We then computed the DSC between the T2-based manual contours and propagated CT-based manual contours. Note that the DSC is biased toward the proposed and structure-guided methods. We also computed the normalised mutual information (NMI) between the T2 and registered CT images to present a surrogate independent measure (as the LNCC was used as similarity measure for the T2 and CT channels during the registration process).

#### 2.4.2. Evaluation of the segmentation and CT synthesis accuracies.

The performance of the joint iterative segmentation and image synthesis framework was compared with reference data for the 15 subjects following a leave-one-out cross-validation strategy. Seven iterations of the pipeline were computed. For each subject, a water-only pseudo CT was also included to set the results in perspective. It was generated by segmenting the body contour from the CT image registered to the T2 image setting a threshold at 500 HU, filling the resulting volume, and assigning it an intensity value of 0 HU.

Both the segmentation and CT synthesis accuracies were evaluated to assess the overall performance of the method and determine the optimal number of iterations.

##### Segmentation accuracy.

The segmentation accuracy was assessed by computing the fuzzy DSC between the manual and probabilistic atlas-based segmentations, and the modified Hausdorff distance (MHD) (Dubuisson and Jain 1994) between the manual and categorical atlas-based segmentations for the different organs considered (bladder, prostate, rectum, and left (L) and right (R) femur heads). The MHD is defined as

8 \begin{eqnarray*}\text{MHD}=\max \left(\frac{1}{{{N}_{a}}}\underset{a\in A}{\sum}\,d(a,B),\frac{1}{{{N}_{b}}}\underset{b\in B}{\sum}\,d(b,A)\right)\end{eqnarray*}

where the distance between two points *a* and *b* is defined as the Euclidean distance $d(a,b)=\parallel a-b\parallel $ and the distance between a point *a* and a set of points $B=\left\{{{b}_{1}},...,{{b}_{{{N}_{b}}}}\right\}$ is defined as $d(a,B)={{\min}_{b\in B}}\parallel a-b\parallel $. Note that we compute this distance on the boundary of a set rather than the set itself.

##### CT synthesis accuracy.

The mean absolute error (MAE) and the mean error (ME) were calculated for every subject between the reference CT non-rigidly aligned to the MR (${{R}^{\text{CT}}}$) and each of the pseudo CTs (${{I}^{\text{pCT}}}$) in a ROI comprising *V* voxels as follows:

9 \begin{eqnarray*}\begin{array}{*{35}{l}} \text{MAE} &amp; =\frac{1}{V}\underset{x}{\sum}\,|{{I}^{\text{pCT}}}(x)-{{R}^{\text{CT}}}(x)| \end{array}\end{eqnarray*}

10 \begin{eqnarray*}\begin{array}{*{35}{l}} \text{ME} &amp; =\frac{1}{V}\underset{x}{\sum}\,\left({{I}^{\text{pCT}}}(x)-{{R}^{\text{CT}}}(x)\right). \end{array}\end{eqnarray*}

Two ROIs were considered: within the external contour and in the bone region (manually delineated on the CT image).

#### 2.4.3. Statistical significance.

The paired one-tailed Wilcoxon signed-rank test, with a 5% significance level, was used to assess the statistical significance of the improvement observed between the different registration strategies used to build the multi-atlas database, and between two iterations of the proposed joint segmentation and image synthesis framework.

#### 2.4.4. Dosimetric evaluation.

Once the optimal number of iterations was defined, dose calculations were performed using the RayStation treatment planning system to assess the applicability of the proposed framework for RTP. Doses were also calculated for the water-only pseudo CT (HU  =  0). The original clinical plans were copied onto the pseudo CTs and doses were re-calculated using the original planning parameters. We compared the cumulative dose volume histograms (DVH) obtained for the pseudo CTs to the DVH obtained for the reference CT image in the planning target volume (PTV) located in the prostate, and in the OARs. The same contours were used for the pseudo and reference CT images.

For all the subjects, dose differences were evaluated for several DVH points: ${{D}_{98 \% }}$, *D*_mean_ and ${{D}_{2 \% }}$ for the PTV, and *D*_mean_ and ${{D}_{2 \% }}$ for the OARs (bladder, rectum and femur heads), following the recommendations described in the ICRU Report 83 (2010). *D*_x_ is the dose given to x% of the structure volume and *D*_mean_ is the mean dose given to the evaluated volume.

## 3. Results

### 3.1. Multi-modal registration for atlas database building

**Figure 3.**
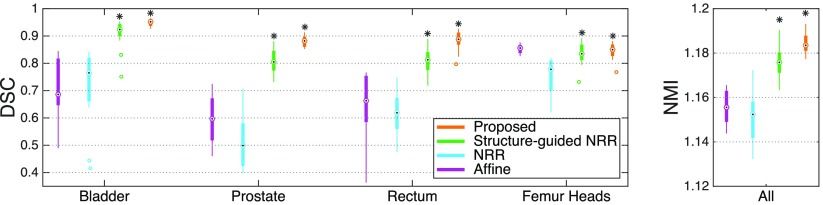
Comparison of different multi-modal T2-CT registration strategies used to build the multi-atlas database. The boxplots display the median, lower and upper quartiles, and minimum and maximum of the DSC calculated between the T2-based and propagated CT-based manual segmentations (left), and of the NMI computed between the T2 and registered CT images (right). The stars indicate a significant improvement between the current and previous strategies.

Boxplots showing the performance of the different registration strategies used to align atlas T2 and CT images are displayed in figure 3. After the affine alignment, bone structures appear to be well aligned (figure 3, left). They should remain well aligned after the non-rigid step, and the DSC for all the organs, as well as the NMI, should increase, but this is not the case when no structures are used to guide the registration (NRR in figure 3). Using structures to guide and constrain the non-rigid registration improves both the segmentation overlap and the similarity between T2 and registered CT images. The proposed method further increases the DSC and NMI.

### 3.2. Segmentation and CT synthesis accuracy

**Figure 4.**
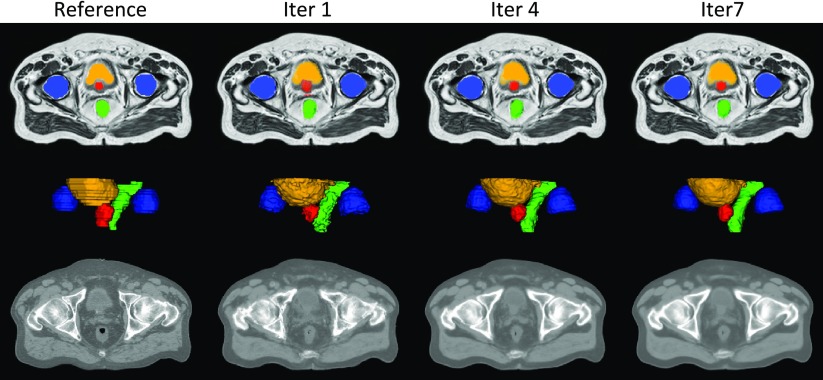
The probabilistic (top) and categorical (middle) segmentations of the prostate (red), bladder (orange), rectum (green) and femur heads (blue), and the pseudo CTs (bottom) were obtained from the T1-weighted and T2-weighted MR images of the patient. Note how the smoothness of the segmentations and sharpness of the pseudo CTs increase with the number of iterations.

An example of reference data and results obtained after the first, fourth and seventh iterations of the proposed framework are displayed in figure 4.

**Figure 5.**
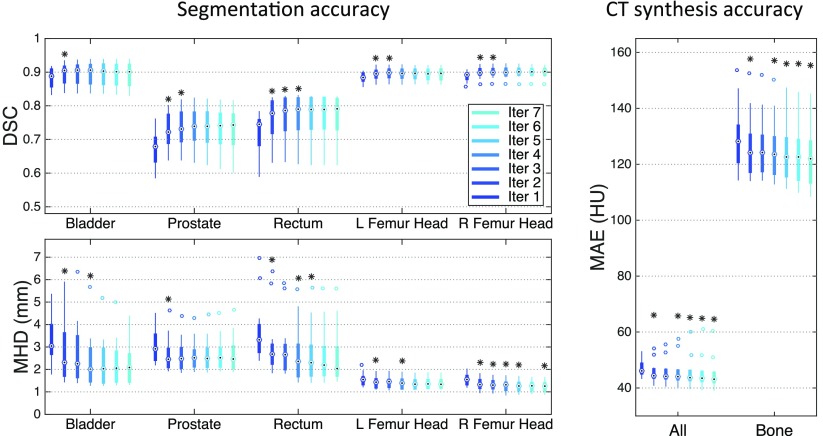
Boxplots displaying the median, lower and upper quartiles, and minimum and maximum of the DSC calculated between the manual and probabilistic atlas-based segmentations (top left); the MHD calculated between the manual and categorical atlas-based segmentations (bottom left); and the MAE computed between the reference and pseudo CTs (right). The stars indicate a significant improvement between the current and previous iterations.

The DSC results displayed in figure 5 show a statistically significant improvement for all the organs considered between the first and second iterations. Although the DSC does not always significantly improve after the second iteration, we observed an increase in the segmentation smoothness when we kept iterating, as illustrated in figure 4. Regarding the MHD results, a statistically significant improvement was found for all the organs considered between the first and second iterations, and for all the OARs between the third and fourth iterations.

In both the full image and the bone region, the MAE showed a significant decrease in CT synthesis error between most iterations (figure 5).

**Figure 6.**
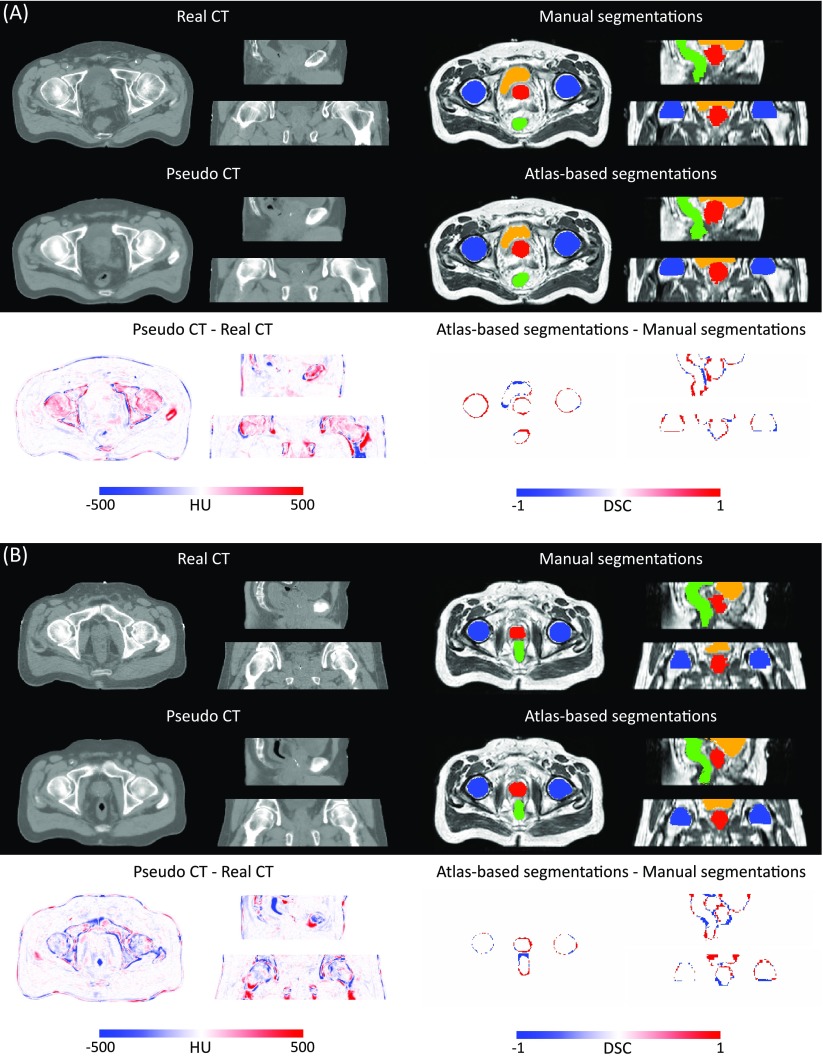
Categorical segmentations of the prostate (red), bladder (orange), rectum (green) and femur heads (blue), pseudo CT obtained from the T1-weighted and T2-weighted MR images of the patient after four iterations of the proposed joint iterative segmentation and image synthesis framework, and difference images for subjects with the best (top) and worst (bottom) results.

Based on these results, and as a compromise between accuracy and computation complexity, four was found to be the optimal number of iterations. Segmentations and pseudo CT obtained after the fourth iteration for the subjects with the best and worst results are displayed in figure 6. DSC and MHD results for the first and fourth iterations are summarised in table 1. MAE and ME results for the first and fourth iterations, as well as for the water-only pseudo CT, are summarised in table 2. The synthesis error obtained with the pseudo CTs generated by the proposed approach is half the error obtained with the water-only pseudo CT. The low ME values demonstrate an absence of bias in the proposed methodology.

**Table 1.** Average  ±  standard deviation of the DSC and MHD obtained for 15 subjects after the first and fourth iterations of the joint iterative segmentation and image synthesis framework.

		Iter 1	Iter 4	Improvement (%)
	Bladder	$0.88\pm 0.03$	$0.90\pm 0.03$	1.6^a^
	Prostate	$0.67\pm 0.05$	$0.73\pm 0.06$	9.2^a^
DSC	Rectum	$0.72\pm 0.06$	$0.77\pm 0.06$	7.2^a^
	LFemurHead	$0.88\pm 0.01$	$0.89\pm 0.02$	1.2^a^
	RFemurHead	$0.89\pm 0.01$	$0.90\pm 0.01$	1.5^a^

	Bladder	$3.34\pm 0.98$	$2.35\pm 1.14$	42.3^a^
	Prostate	$3.10\pm 0.77$	$2.63\pm 0.67$	17.5^a^
MHD (mm)	Rectum	$3.63\pm 1.29$	$2.62\pm 1.19$	38.6^a^
	LFemurHead	$1.56\pm 0.26$	$1.38\pm 0.22$	12.8^a^
	RFemurHead	$1.55\pm 0.24$	$1.29\pm 0.23$	20.4^a^

a Significant improvement.

**Table 2.** Average  ±  standard deviation of the MAE and ME obtained for 15 subjects for the water-only pseudo CT, and after the first and fourth iterations of the joint iterative segmentation and image synthesis framework.

		Water-only	Iter 1	Iter 4	Improvement Iter 1 → 4 (%)
	All	$89.7\pm 2.8$	$47.0\pm 2.9$	$45.7\pm 4.6$	2.9^a^
MAE (HU)	Bone	$264.8\pm 30.5$	$129.1\pm 10.7$	$125.1\pm 10.3$	3.2^a^

	All	$-16.0\pm 9.8$	$-0.5\pm 6.7$	$-1.6\pm 7.7$	
ME (HU)	Bone	$-261.7\pm 31.9$	$-16.3\pm 32.6$	$-11.1\pm 32.8$	

a Significant improvement.

### 3.3. Dosimetry calculations

**Figure 7.**
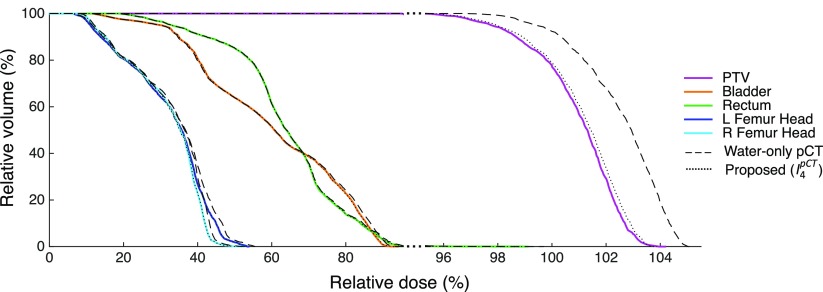
DVHs obtained for the reference CT (solid lines), water-only pseudo CT (dashed lines) and pseudo CT obtained after the fourth iteration of the proposed framework (dotted lines) in the PTV and OARs for a representative subject. Note the change in scale on the *x*-axis when the relative dose is higher than 95%.

**Figure 8.**
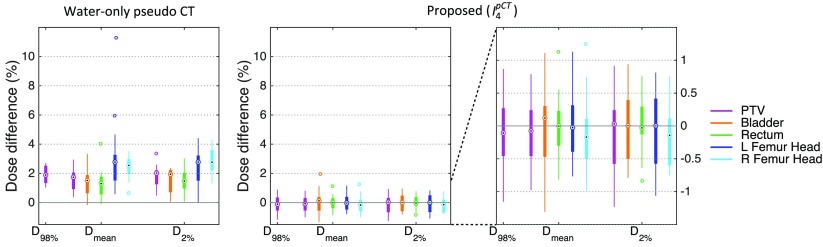
Boxplots of the dose differences evaluated at several DVH points for 15 subjects for the water-only pseudo CT and the pseudo CT obtained after the fourth iteration of the proposed framework. Note that the first two plots share the same scale while the last plot zooms in on the results obtained with the proposed method.

Dose calculations were performed for the water-only pseudo CTs and for the pseudo CTs obtained after four iterations. The DVHs displayed in figure 7 for a representative subject show a close agreement between the doses calculated from the reference and proposed pseudo CTs while larger differences are observed between the reference and water-only pseudo CTs. Boxplots showing the dose differences evaluated for several DVH points are displayed in figure 8. With the proposed method, for all the DVH points considered, the dose difference is on average less than  ±0.15% for the PTV and all the OARs. With the water-only pseudo CT, a systematic bias is observed. For most DVH points, the dose difference is on average between 1.5% and 3%.

## 4. Discussion

In this paper, we presented a joint segmentation and CT synthesis framework to automatically generate accurate pseudo CT images and organ contours in the pelvic region for MRI-only RTP.

The method relies on a database of multiple atlases, each atlas consisting of a T2-weighted MR image, a T1-weighted MR image, a CT image and a segmented image obtained by manually contouring the T2-weighted MR image. Both the T1-weighted MR image and the CT image have to be registered to the T2-weighted MR image. To align T1-weighted and T2-weighted MR images, a structure-guided strategy, as proposed by Rivest-Hénault *et al* (2013), provides acceptable results. Because of differences in acquisition setting and morphological changes such as different bladder or rectum filling, registering CT and T2-weighted MR images is more challenging. To improve the T2-CT registration, we proposed to combine structure-guided registration and image synthesis. Pseudo CT and pseudo T2 images were generated and used together with the original CT and T2-weighted MR images, and the segmented images, as inputs for a multi-channel non-rigid registration. Although many methods exist to synthesise CT from MR images, such as the atlas-based methods (Gudur *et al* 2014, Uh *et al* 2014, Burgos *et al* 2015, Dowling *et al* 2015, Sjölund *et al* 2015), to our knowledge this is the first time that an atlas-based method was used to synthesise MR from CT images (pT2 in section 2.3.2 and figure 2). This task is more challenging as less information regarding the soft-tissues is available in a CT image than in a structural MR image. With the proposed registration strategy, we visually observed that the T2 and CT images were better aligned, which was supported by the NMI and segmentation overlap results displayed in figure 3.

In the proposed iterative framework, a set of probabilistic segmentations and pseudo CT images is jointly generated from the target subject’s MR images by registering the atlases to the target, and fusing the atlas segmentations and CT images according to the similarity between the target and each atlas. Solving the segmentation and synthesis tasks simultaneously results in having the solutions in agreement, but also the joint estimation aids in improving the accuracy of each aspect, as seen in figure 5, where we observe that an increase in segmentation overlap corresponds to a decrease in CT synthesis error.

Two structural MR images (T2-weighted and T1-weighted) were used as inputs for the method as combining contrasts increases the synthesis accuracy, as previously shown in Burgos *et al* (2015a). A single sequence or any combination of sequences providing enough structural information and structural contrast could be used as inputs.

Registration is an important step to generate accurate pseudo CTs and segmentations. In this work we used the NiftyReg^9^9https://sourceforge.net/projects/niftyreg/ package for both the intra-subject and inter-subject registrations. In both cases, the quality of the alignments was checked visually for several sets of parameters to select the optimal one. Note that NiftyReg is a fast, general purpose registration package and that the same result accuracy cannot be guaranteed using other registration packages, but could probably be improved using task-specific methods.

The performance of the proposed joint iterative segmentation and image synthesis framework was compared with reference data for 15 subjects following a leave-one-out cross-validation strategy. We first determined the optimal number of iterations by assessing the segmentation and CT synthesis accuracy after each iteration. Four was found to be a good compromise between accuracy and computational complexity. We then performed dose calculations to assess the applicability of the proposed framework for RTP and found that on average for all the DVH points considered, the dose difference was less than  ±0.15% for the PTV and all the OARs. These results fall within the acceptable deviation range specified in Korsholm *et al* (2014), i.e. a 2% deviation in PTV coverage for 95% of the patients. To set the results in perspective, we generated pseudo CTs with a uniform intensity of 0 HU applied to the body. With the water-only pseudo CT, the dose was systematically overestimated, with average dose differences for most DVH points comprised between 1.5% and 3%.

The first iteration of the proposed framework is similar to the method developed by Dowling *et al* (2015) as both the inter-subject mapping and fusion steps are based on the MR images only. Results displayed in tables 1 and 2 show the benefits of the iterative framework as both the segmentation and CT synthesis accuracies are improved.

When evaluating the segmentation accuracy obtained with their method, Dowling *et al* (2015) reported mean DSCs of 0.80, 0.86 and 0.84 for the prostate, bladder and rectum, respectively. Wong *et al* (2015) assessed the performance of several multi-atlas segmentation methods. With their recommended setting, the median DSC for the prostate, bladder, rectum and femurs was 0.84, 0.90, 0.77 and 0.95, respectively. These results are not directly comparable to the ones obtained in this work as we used lower resolution images (5 mm slice thickness versus 2 mm in Dowling *et al* (2015) and 2.5 mm in Wong *et al* (2015)), and DSC is known to highly correlate with image resolution. However, taking into account this lower resolution, we obtained comparable results with mean DSCs of 0.73, 0.90, 0.77 and 0.90. Dowling *et al* (2015) also reported results for the mean absolute surface distance, equivalent to the MHD used in this work, of 5.10 mm, 2.37 mm and 1.45 mm for the bladder, rectum and femur heads, respectively. As the MHD is measured in millimetres, results are more comparable between resolutions. Given this, we obtained comparable results with mean MHD of 2.35 mm, 2.62 mm and 1.33 mm for the bladder, rectum and femur heads, respectively.

Regarding the CT synthesis, the proposed method generates pseudo CTs with similar accuracy as previously reported for the pelvic region, even though they were obtained from lower resolution MR images. Dowling *et al* (2015) reported an average MAE of $40.5\pm 8.2$ HU while we obtained an average MAE of $45.7\pm 4.6$ HU.

Even though different DVH points were analysed, dosimetric results appear to be of the same order of magnitude as the one presented in Dowling *et al* (2015) and Arabi *et al* (2016). Dowling *et al* (2015) reported average DVH parameter differences of  −0.3% for ${{D}_{95 \% }}$, −0.5% for ${{D}_{50 \% }}$ and  −0.4% for ${{D}_{5 \% }}$, while Arabi *et al* (2016) reported average differences comprised between  −0.3% and 0.5% depending on the organs considered (prostate, bladder, rectum and femur heads) for DVH points between ${{D}_{100 \% }}$ and ${{D}_{0 \% }}$. With the proposed approach, we obtained average differences of  −0.14% in the PTV for ${{D}_{98 \% }}$, and between  −0.14% and 0.05% in the PTV, bladder, rectum and femur heads for *D*_mean_ and ${{D}_{2 \% }}$.

In this work, the treatment plans were optimised on the reference CTs to create a ground truth and re-calculated for the pseudo CTs to assess if the pseudo CTs were accurate enough to be used for dose calculations. However, in an MRI-only workflow, the plans would be optimised on the pseudo CTs. Future work will consist of optimising the plans on the pseudo CTs and re-calculating them for the reference CTs to assess if differences exist between these two optimisation and evaluation strategies, and if so quantify them. Note however that Korsholm *et al* (2014) observed no significant change between these two strategies.

The clinical contours obtained by manually segmenting the reference CT image were applied to both the reference and pseudo CT images for the dosimetric evaluation. In the future, we plan to compare the plans obtained from the reference CT image and manual segmentations to the plans obtained from the pseudo CTs and segmentations generated by the proposed method on a larger dataset, and thus be able to assess the suitability of the whole framework for RTP. Before performing this evaluation, we will ask three observers to contour the organs of interest. This will allow us to generate a gold standard contour for each organ, for example using majority voting to combine the observer contours, and thus to assess inter-observer variability.

Only contours of the whole bladder and rectum were available in this study, and not the contours of the walls, which are the actual structures of interest. However, the method could be applied to contours of these structures’ walls if these were the ones available.

Finally, as future work, the probabilistic property of the segmentations obtained with the proposed framework could be used to automatically define margins when contouring organs and improve MR-based RTP.

## 5. Conclusion

This paper presents a joint segmentation and CT synthesis framework for MRI-only RTP able to automatically generate accurate pseudo CT images and organ contours in the pelvic region. Solving the segmentation and synthesis tasks simultaneously results in not only having the solutions in agreement, but the joint estimation aids in improving the accuracy of each aspect. The high segmentation and CT synthesis accuracies, and the low dosimetric errors suggest that the proposed framework could facilitate the clinical deployment of MR-linac devices.

## References

[pmbaa66bfbib001] Arabi H, Koutsouvelis N, Rouzaud M, Miralbell R, Zaidi H (2016). Atlas-guided generation of pseudo-CT images for MRI-only and hybrid PET-MRI-guided radiotherapy treatment planning. Phys. Med. Biol..

[pmbaa66bfbib002] Bai W, Shi W, O’Regan D P, Tong T, Wang H, Jamil-Copley S, Peters N S, Rueckert D (2013). A probabilistic patch-based label fusion model for multi-atlas segmentation with registration refinement: application to cardiac MR images. IEEE Trans. Med. Imaging.

[pmbaa66bfbib003] Burgos N, Cardoso M J, Guerreiro F, McClelland J, Knopf A, Ourselin S (2016a). Simultaneous organ-at-risk segmentation and CT synthesis in the pelvic region for MRI-only radiotherapy treatment planning.

[pmbaa66bfbib004] Burgos N (2015). Robust CT synthesis for radiotherapy planning: application to the head & neck region.

[pmbaa66bfbib005] Burgos N, Cardoso M J, Thielemans K, Modat M, Dickson J, Schott J M, Atkinson D, Arridge S R, Hutton B F, Ourselin S (2015a). Multi-contrast attenuation map synthesis for PET/MR scanners: assessment on FDG and Florbetapir PET tracers. Eur. J. Nucl. Med. Mol. Imaging.

[pmbaa66bfbib006] Burgos N (2014). Attenuation correction synthesis for hybrid PET-MR scanners: application to brain studies. IEEE Trans. Med. Imaging.

[pmbaa66bfbib007] Burgos N, Guerreiro F, McClelland J, Nill S, Dearnaley D, deSouza N, Oelfke U, Knopf A, Ourselin S, Cardoso M J (2016b). Joint segmentation and CT synthesis for MRI-only radiotherapy treatment planning.

[pmbaa66bfbib008] Cabezas M, Oliver A, Lladó X, Freixenet J, Cuadra M B (2011). A review of atlas-based segmentation for magnetic resonance brain images. Comput. Methods Programs Biomed..

[pmbaa66bfbib009] Cachier P, Bardinet E, Dormont D, Pennec X, Ayache N (2003). Iconic feature based nonrigid registration: the PASHA algorithm. Comput. Vis. Image Underst..

[pmbaa66bfbib010] Cao X, Gao Y, Yang J, Wu G, Shen D (2016). Learning-based multimodal image registration for prostate cancer radiation therapy.

[pmbaa66bfbib011] Chen M, Jog A, Carass A, Prince J L (2015). Using image synthesis for multi-channel registration of different image modalities. SPIE Medical Imaging.

[pmbaa66bfbib012] Dowling J A, Lambert J, Parker J, Salvado O, Fripp J, Capp A, Wratten C, Denham J W, Greer P B (2012). An atlas-based electron density mapping method for magnetic resonance imaging (MRI)-alone treatment planning and adaptive MRI-based prostate radiation therapy. Int. J. Radiat. Oncol. Biol. Phys..

[pmbaa66bfbib013] Dowling J A (2015). Automatic substitute computed tomography generation and contouring for magnetic resonance imaging (MRI)-alone external beam radiation therapy from standard MRI sequences. Int. J. Radiat. Oncol. Biol. Phys..

[pmbaa66bfbib014] Dubuisson M P, Jain A K (1994). A modified hausdorff distance for object matching.

[pmbaa66bfbib015] Gudur M S R, Hara W, Le Q T, Wang L, Xing L, Li R (2014). A unifying probabilistic Bayesian approach to derive electron density from MRI for radiation therapy treatment planning. Phys. Med. Biol..

[pmbaa66bfbib016] Heckemann R A, Hajnal J V, Aljabar P, Rueckert D, Hammers A (2006). Automatic anatomical brain MRI segmentation combining label propagation and decision fusion. NeuroImage.

[pmbaa66bfbib017] Iglesias J E, Konukoglu E, Zikic D, Glocker B, Van Leemput K, Fischl B (2013). Is synthesizing MRI contrast useful for inter-modality analysis?.

[pmbaa66bfbib018] Izquierdo-Garcia D, Catana C (2016). MR imaging–guided attenuation correction of PET data in PET/MR imaging. PET Clinics.

[pmbaa66bfbib019] Klein S, van der Heide U A, Lips I M, van Vulpen M, Staring M, Pluim J P (2008). Automatic segmentation of the prostate in 3D MR images by atlas matching using localized mutual information. Med. Phys..

[pmbaa66bfbib020] Korsholm M E, Waring L W, Edmund J M (2014). A criterion for the reliable use of MRI-only radiotherapy. Radiat. Oncol..

[pmbaa66bfbib021] Modat M, Cash D M, Daga P, Winston G P, Duncan J S, Ourselin S (2014). Global image registration using a symmetric block-matching approach. J. Med. Imaging.

[pmbaa66bfbib022] Modat M, Ridgway G R, Daga P, Cardoso M J, Ashburner J, Ourselin S (2012). Parametric non-rigid registration using a stationary velocity field.

[pmbaa66bfbib023] Modat M, Ridgway G R, Taylor Z A, Lehmann M, Barnes J, Hawkes D J, Fox N C, Ourselin S (2010). Fast free-form deformation using graphics processing units. Comput. Methods Programs Biomed..

[pmbaa66bfbib024] Rasch C, Steenbakkers R, van Herk M (2005). Target definition in prostate, head, and neck. Semin. Radiat. Oncol..

[pmbaa66bfbib025] Report 83 (2010). Prescribing, recording, and reporting photon-beam intensity-modulated radiation therapy (IMRT): contents. J. ICRU.

[pmbaa66bfbib026] Rivest-Hénault D, Greer P, Fripp J, Dowling J (2013). Structure-guided nonrigid registration of CT–MR pelvis scans with large deformations in MR-based image guided radiation therapy.

[pmbaa66bfbib027] Roy S, Carass A, Jog A, Prince J L, Lee J (2014). MR to CT registration of brains using image synthesis. SPIE Medical Imaging.

[pmbaa66bfbib028] Sjölund J, Forsberg D, Andersson M, Knutsson H (2015). Generating patient specific pseudo-CT of the head from MR using atlas-based regression. Phys. Med. Biol..

[pmbaa66bfbib029] Tustison N J, Avants B B, Cook P A, Zheng Y, Egan A, Yushkevich P A, Gee J C (2010). N4ITK: improved N3 bias correction. IEEE Trans. Med. Imaging.

[pmbaa66bfbib030] Uh J, Merchant T E, Li Y, Li X, Hua C (2014). MRI-based treatment planning with pseudo CT generated through atlas registration. Med. Phys..

[pmbaa66bfbib031] Wang H, Suh J W, Das S R, Pluta J B, Craige C, Yushkevich P A (2013). Multi-atlas segmentation with joint label fusion. IEEE Trans. Pattern Anal. Mach. Intell..

[pmbaa66bfbib032] Wang Z, Bovik A C, Sheikh H R, Simoncelli E P (2004). Image quality assessment: from error visibility to structural similarity. IEEE Trans. Image Process..

[pmbaa66bfbib033] Wong W K, Leung L H, Kwong D L (2015). Evaluation and optimization of the parameters used in multiple-atlas-based segmentation of prostate cancers in radiation therapy. Br. J. Radiol..

[pmbaa66bfbib034] Zadeh L A (1965). Fuzzy sets. Inf. Control.

